# An inhibitory receptor of VLRB in the agnathan lamprey

**DOI:** 10.1038/srep33760

**Published:** 2016-10-20

**Authors:** Fenfang Wu, Liyong Chen, Yong Ren, Xiaojing Yang, Tongzhou Yu, Bo Feng, Shangwu Chen, Anlong Xu

**Affiliations:** 1 State Key Laboratory of Biocontrol, Guangdong Province Key Laboratory for Pharmaceutical Functional Genes, Department of Biochemistry, School of Life Sciences, Sun Yat-Sen (Zhongshan) University, Guangzhou 510275, P. R. China; 2 Guangdong Province Key Laboratory for Medical Molecular Diagnostics, China-America Cancer Research Institute, Dongguan Scientific Research Center, Guangdong Medical University, Dongguan 523808, P. R. China; 3 Beijing University of Chinese Medicine, Beijing, 100029, P. R. China

## Abstract

Lamprey, the primitive jawless vertebrate, uses variable lymphocyte receptor (VLR) as alternative adaptive immune system instead of immunoglobulin (Ig)-based receptors used in jawed vertebrates. In the present study, we characterized a potential inhibitory receptor of VLRB from leucocytes in lamprey. It is a novel ITIM-containing IgSF protein and was therefore named as NICIP. NICIP has two Ig-like domains in extracellular region, a transmembrane domain and two classical ITIM motifs in cytoplasmic domain. It is mainly expressed on the surface of granulocytes and monocytes and can interact with VLRB. In transiently transfected HEK293T cells, it was confirmed again that it could interact with VLRB and the two phosphorylated ITIM motifs could recruit SHP-1 and SHP-2. These results imply that NICIP may play a role as a potential inhibitory receptor of VLRB and involve in negative regulation of immune response mediated by VLRB.

Jawed vertebrates and jawless vertebrates possess distinct adaptive immune systems^1^,^2^,^3^. Jawless vertebrates lampreys and hagfish use leucine-rich-repeat (LRR)-based receptors, called variable lymphocyte receptors (VLRs), for antigen recognition instead of the immunoglobulin (Ig)-based receptors used in jawed vertebrates^4^,^5^. Three VLR genes (VLRA, VLRB, and VLRC) have been identified in lampreys and hagfish, and are expressed on three distinct lymphocytes lineages^6^,^7^,^8^. VLRA^+^ cells and VLRC^+^ cells are T-cell-like and develop in a thymus-like lympho-epithelial structure, termed thymoids^9^. VLRB^+^ cells are B-cell-like, develop in hematopoietic organs, and differentiate into “VLRB antibody”-secreting plasma cells^10^.

Though lamprey lacks Ig-based adaptive immune system, several immunoglobulin superfamily (IgSF) molecules containing typical Ig-like domain were described in these jawless vertebrates, such as Netrin-like protein (GenBank accession no. AF129475), NICIR1, 2, 3^11^,^12^, APAR^13^, TCR-like^14^, IGSF1, and VpreB-like molecules^15^.

In this article, we identified a novel IgSF protein on the surface of leukocytes in lamprey. It contains two typical immuno-receptor tyrosine-based inhibitory motifs (ITIM), which were characterized by conserved sequence of amino acids (S/I/V/LxYxxI/V/L). Thus, this novel ITIM-containing IgSF protein was termed as NICIP. We also found that NICIP could interact with secreted VLRB and recruit SHP-1 and SHP-2 through phosphorylated ITIM to function as inhibitory receptor of VLRB.

## Results

### Identification and phylogenetic analysis of *NICIP* gene in lamprey

A single EST homologous to *NICIP* was found among the extensive EST sequences from the cDNA library of lamprey blood cells. By 3′-RACE and 5′-RACE, a full-length NICIP cDNA with 2425-bp nucleotide was obtained. It contains a 975-bp open reading frame (ORF) encoding 324 amino acid residues with a predicted molecular weight of about 35 kDa. The NICIP cDNA sequence was submitted to GenBank database with the accession number of KM595284.1.

**Figure 1:**
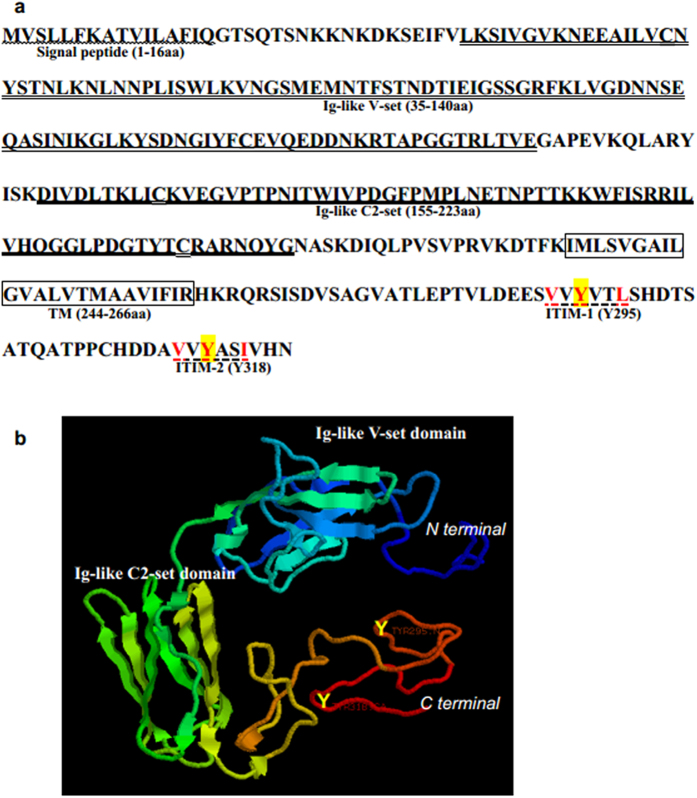
Identification and prediction of the conserved domains of NICIP in Lamprey. (**a**) Sequences and domains of NICIP. Wavy lines: Signal peptide, Double underline: Ig-like V-set, Underline: Ig-like C2-set, Character border: TM (Transmembrane) domain, Dashed underline: ITIMs. Red is key amino acid and yellow is potential tyrosine phosphorylation sites in ITIM. (**b**) Three dimensional structure of NICIP. Y means tyrosine in C-terminal of NICIP.

The NICIP contains five regions; Ig-like V-set domain, Ig-like C2-set domain, transmembrane region and the two potential ITIM motifs with adjoining amino acid residues. The residues sequences are close to the consensus VxYxxL/I/V (Fig. 1a). Protein structure prediction of NICIP also showed that it had typical Ig-like domains and an intracellular tail containing two potential tyrosine phosphorylation sites (Fig. 1b).

### Tissue distribution of NICIP

**Figure 2:**
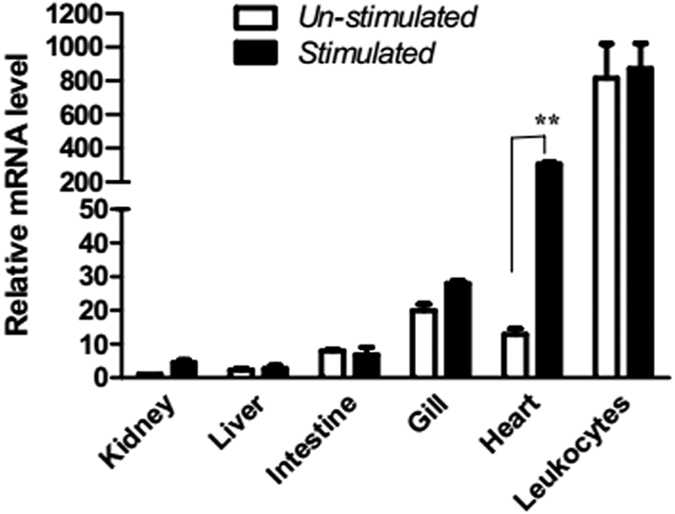
Real-time PCR analysis of *NICIP* mRNA expressed in adult tissues. The relative expression quantity of *NICIP* mRNA in other tissues compared to kidney from un-stimulated lamprey was calculated. All tissues samples were performed in triplicates. **P < 0.01.

The expression of lamprey *NICIP* mRNA in various tissues was examined using Real-time PCR. The highest level of expression of NICIP was in leukocytes, followed by heart, gill, and intestine. A small amount of expression was in liver and kidney. The expression levels of NICIP in heart were significantly increased after LPS stimulation (Fig. 2).

### The cellular localization and the interaction of NICIP and VLRB protein

**Figure 3:**
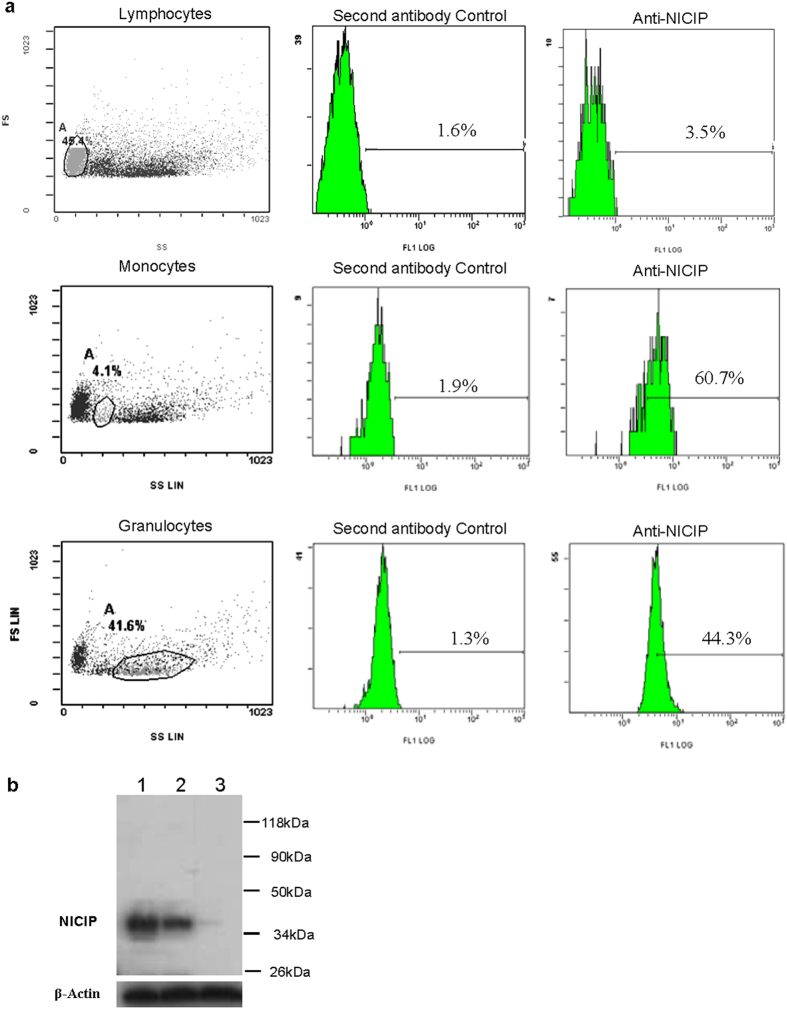
Distribution of NICIP on the surface of different leukocyte subsets. The experiments were done three times independently, and the representative result is shown. (**a**) Distribution of NICIP on the surface of leukocyte subsets by flow cytometry analysis. (**b**) western blotting assays. According to the amino acid sequences, the predicted molecular weight of NICIP is about 36 kDa. Lane 1, monocyte lysate. Lane 2, granulocyte lysate. Lane 3, lymphocyte lysate.

**Figure 4:**
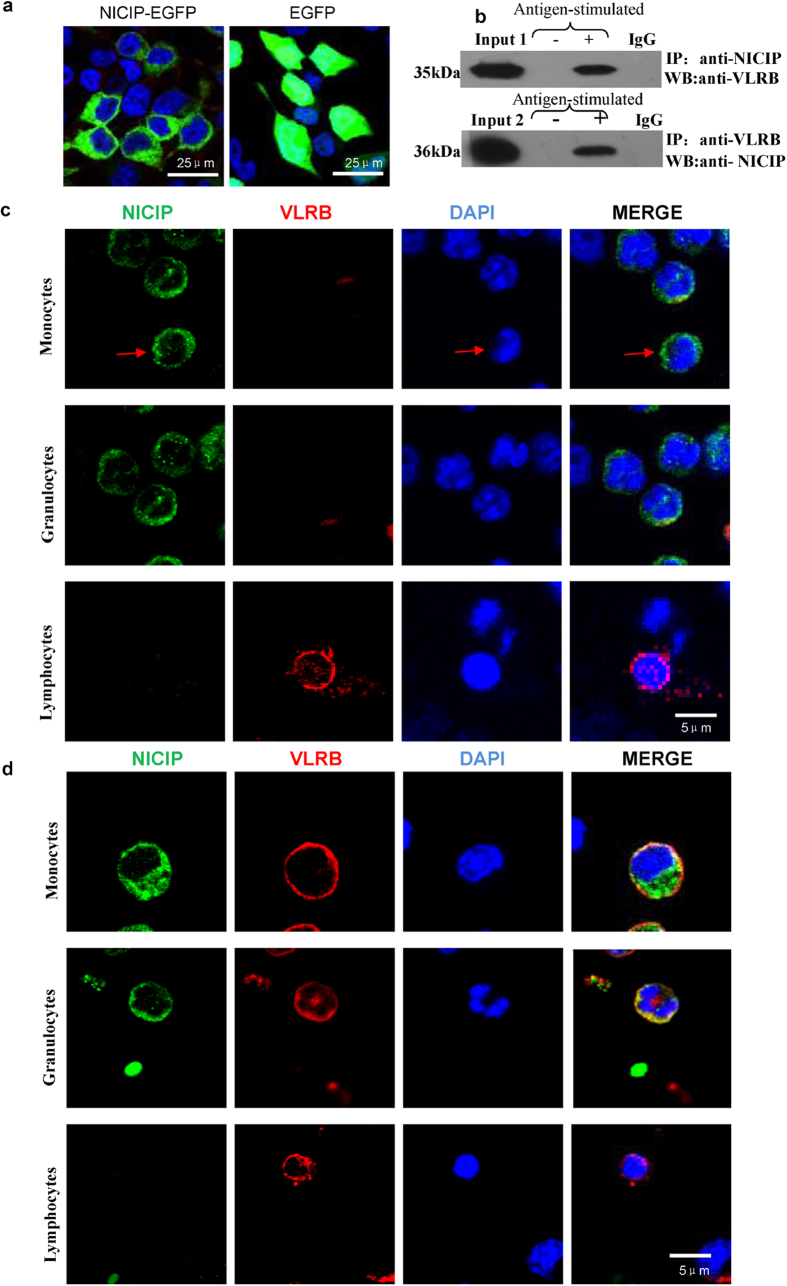
The interaction between NICIP on the membrane of lamprey leukocytes and free secreted-type VLRB in the antisera. (**a**) Membrane localization of NICIP-EGFP fusion protein in transiently transfected 293T cells. (**b**) The interaction of VLR and NICIP by co-immunoprecipitation analysis. Lamprey leukocytes were pre-incubated using lamprey anti-LPS antisera with or without LPS antigen. Input 1 was antisera. Input 2 was leukocytes lysate. (**c**) Localization of NICIP on the surface of lamprey granulocytes and monocytes (red arrow) and localization of membranous-type VLRB on the surface of lamprey lymphocytes. (**d**) Interaction between free secreted-type VLRB and NICIP on the surface of lamprey granulocytes and monocytes but not on lymphocytes under the condition of pre-stimulating by antisera and antigen.

By flow cytometry, after incubating lamprey leukocytes and anti-NICIP rabbit antibodies and FITC-labeled anti-rabbit goat antibodies, we further found that NICIP expressed in 58.8% monocytes, 43% granulocytes and only 1.9% lymphocytes (Fig. 3a). This result was consistent with western blotting assays (Fig. 3b). In transiently transfected 293T cells, NICIP-GFP fusion protein was expressed and mainly located on the cell membrane (Fig. 4a). In order to verify the interaction of NICIP and VLRB, lamprey leucocytes were pretreated with lamprey anti-LPS antisera with or without LPS antigen. Then, the cells lysates were immunoprecipitated with mouse anti-VLRB monoclonal antibodies or rabbit anti-NICIP polyclonal antibodies. The anti-NICIP antibodies could co-precipitate a 35 kDa protein which was recognized by anti-VLRB antibody with western blotting. Conversely, a 36 kDa band precipitated by anti-VLRB antibody was recognized by anti-NICIP antibody (Fig. 4b). Furthermore, the cellular localization (Fig. 4c) and the interaction (Fig. 4d) between secreted-type VLRB and NICIP on the surface of lamprey leucocytes pre-stimulated by antisera and corresponding antigen were confirmed by fluorescent confocal analysis.

### Recruitment of SHP-1 and SHP-2 by phosphorylated ITIMs of NICIP

**Figure 5:**
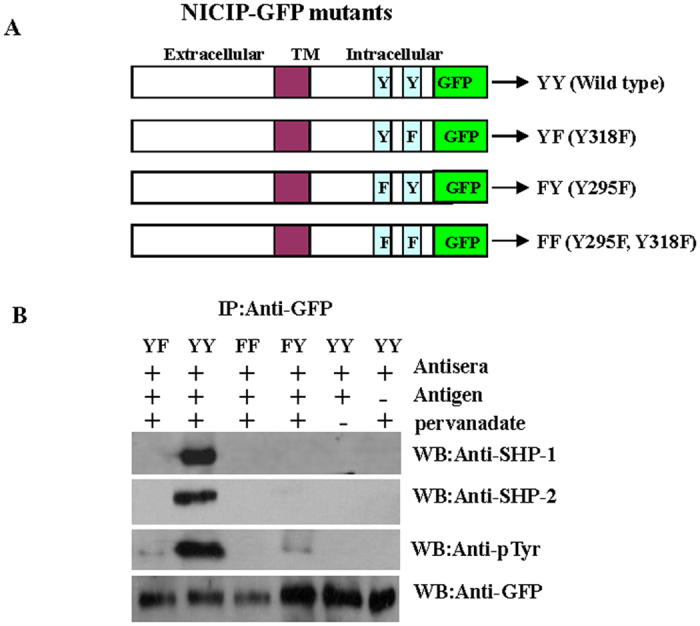
Immunoprecipitation analysis of SHP-1 and SHP-2 association with NICIP. (**A**) Mutants construction of NICIP-GFP fusion protein expression vector. Tyrosines in ITIM of NICIP are indicated by the letter Y, while tyrosines to phenylalanine mutations are indicated by F. TM, transmembrane region. (**B**) 293T cells of overexpressing NICIP-GFP were stimulated using lamprey anti-LPS antisera with or without LPS. Whole cell lysates were immunoprecipitated (IP) with anti-GFP polyclonal antibody, and immunoprecipitates were subjected to western blotting (WB) analysis with anti-SHP-1, anti-SHP-2 and anti-pTyr antibodies. Equal loading was verified by re-probing of the membranes with anti-GFP antibodies.

NICIP has two tyrosines in its two ITIMs in cytoplasmic tail. Phosphorylated tyrosines in these motifs could serve as docking sites for the tyrosine phosphatases SHP-1/2 and/or SHIP-1. Could NICIP then function as an inhibitory receptor, regulating the phosphorylation of an activating counterpart or other proteins associated with monocytes and granulocytes activation? To test this possibility, the transiently transfected 293T cells with different plasmids as pEGFP-NICIP wt (YY), pEGFP-NICIP^Y295F^ (FY), pEGFP-NICIP^Y318F^ (YF) and pEGFP-NICIP^Y295F, Y318F^ (FF) (Fig. 5A) were cultured to a density of 80%. Then, the transfected 293T cells were pre-incubated in lamprey antisera with corresponding antigen or without antigen. Next, the cells were treated with pervanadate as tyrosine phosphatase inhibitors. The cells were then lysed and immunoprecipitated with anti-GFP mouse antibodies. Precipitation products were analyzed by western blotting and showed that the phosphatases, SHP-1 and SHP-2 co-immunoprecipitated with wild type phosphorylated NICIP, but less or not with Y295F-, Y318F- and Y295F-Y318F-NICIP (Fig. 5B) under antigen-stimulated condition.

## Discussion

In human, different classes of Fc receptors for IgG (FcγRs) have been defined, known as FcγRI (CD64), FcγRII (CD32), and FcγRIII (CD16)^16^,^17^. FcγRI, FcγRIIA, FcγRIIC and FcγRIII are activating receptors, while FcγRIIB is inhibitory receptor^18^,^19^. FcγR families play a crucial role in antibody-mediated effector mechanisms. VLRB as the counterpart of IgG are involved in various branches of the defense in adaptive immune system of lamprey, but the receptor of VLRB is not known.

**Figure 6:**
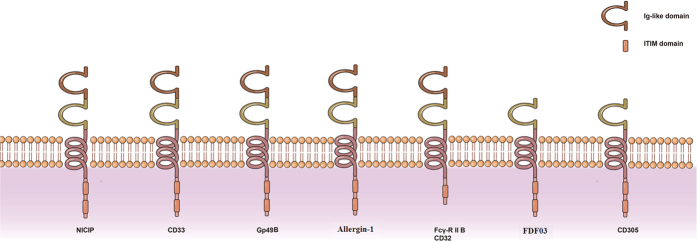
Comparison of the IgSF members containing ITIM. Domains and amino acid sequences for the following proteins were cited from UniProtKB/Swiss-Prot: myeloid cell surface antigen CD33 (Human), P20138.2; leukocyte immunoglobulin-like receptor subfamily B member 4 (Gp49B, Mouse), Q64281; Allergin-1 (Human), Q7Z6M3; low affinity immunoglobulin gamma Fc region receptor II-b (CD32) (Human), P31994; paired immunoglobulin-like type 2 receptor alpha (FDF03) (Human), Q9UKJ1 and leukocyte-associated immunoglobulin-like receptor 1 (CD305) (Human), Q6GTX8.

In this study, we identified a potential inhibitory receptor of VLRB, named NICIP. The receptor belongs to the immunoglobulin superfamily, which contains two Ig-like domains in extracellular region, a transmembrane domain and two potential ITIM motifs in cytoplasmic domain. The two signatures of YxxL/I in ITIMs of NICIP are separated by 17 amino acids, while, the signature of YxxL/I in immunoreceptor tyrosine-based activation motif (ITAM) were typically separated by 6 and 8 amino acids (YxxL/Ix(6–8)YxxL/I). The typical domains of NICIP demonstrate that it may be an inhibitory receptor of IgSF. For the primary sequence of amino acids, NICIP has the lower sequence homology (<25%) with ITIM-containing IgSF proteins from other vertebrates (data not shown), which indicate its evolutionary independence. But taking into account the domain, NICIP is similar to the CD33, Gp49B, and Allergin-1 (Fig. 6). The latter three molecules are members of the IgSF with classical ITIM motif. There are also two tyrosine residues (Y295 and Y318) in NICIP’s ITIM motifs. After being phosphorylated, these tyrosines might function as docking sites for the phosphatases such as SHP-1 and/or SHP-2, enabling NICIP to function as potential inhibitory receptors that might contribute to the regulation of immune functions.

By real time PCR assay, NICIP mainly expressed in lamprey leukocytes. Further analysis by flow cytometry and western blotting showed that it was highly expressed in the granulocytes and monocytes. Which is different from VLR, later mainly expresses in lamprey lymphocytes. Surprisingly, the expression of NICIP in the heart was significantly increased after LPS-stimulated. Our earlier studies reported that CD9^20^ and CD29^21^ expression level was also significantly higher in lamprey heart after LPS-stimulation. In the interstitial areas of human heart, macrophage-like cells were found and could be stained by CD16, CD32, and CD64 mAbs^22^. There was a significant correlation between capillary CD16 expression and heart transplant rejection^23^. CD32 maintains peripheral tolerance *in vivo*, and the reduced expression of inhibitory CD32 contribute to the increased susceptibility to the development of autoantibodies and autoimmune disease^18^. The Arthus reaction in the mouse is the strict requirement for the activation CD16 in initiating inflammation via IgG immune complex, once again limited in its response by the expression of the inhibitory CD32. Given this, the higher expression of NICIP as an inhibitory receptor in heart might be somehow “protecting” the heart from getting involved in a deleterious immune response that could compromise such a noble pumping function.

In 293T cells transfected with pEGFP-N1-NICIP, NICIP located on the membrane of transfected cell. By flow cytometry and laser scanning confocal microscopy analysis, the membrane location of NICIP on lamprey leukocytes was also confirmed. After incubating with the lamprey antisera and antigen, NICIP on the surface of lamprey leukocytes could interact with VLRB in lamprey sera, which demonstrated by laser scanning confocal microscopy analysis. Co-immunoprecipitation and western blotting assay also showed their interaction. These results indicated that NICIP might be a receptor of VLRB.

In order to verify the inhibitory receptor, the tyrosine residues (Y295 and Y318) of each ITIM in NICIP were mutated to phenylalanine, either alone or together. Three mutants and one wild type plasmids were constructed and transfected into 293T cells. In the conditions stimulated by antigen and lamprey sera, wild-type ITIM (Y295-Y318) could recruit SHP-1 and SHP-2. But three mutants Y295F, Y318F and FF showed a consistent reduction of tyrosine phosphorylation by phosphor-tyrosine immunoblot analysis. The three mutants consistently bound less SHP-1 or SHP-2. This suggests that both Y295 and Y318 are phosphorylated and serve to stabilize the interaction of SHP-1, SHP-2 and ITIM motif of NICIP. The difference of tyrosine phosphorylation level between wild type and mutation of ITIM motif indicates that both ITIMs in NICIP contribute to the inhibitory function.

In conclusion, we identified a novel inhibitory immune receptor (named NICIP) that belongs to IgSF from lamprey leukocytes. Its extracellular region has two Ig-like domains. The molecule mainly located on the surface of monocytes and granulocytes and could interact with VLRB in sera. Its intracellular region has two ITIM motifs containing one tyrosine residue Y295 and Y318, respectively. After being stimulated with antigen, the two tyrosine residues are phosphorylated and the two ITIMs could recruit SHP-1 and SHP-2. We assume that NICIP might serve as an inhibitory receptor to negatively regulate the signal transduction mediated by VLRB in lamprey leukocytes.

## Methods

### Ethical statement

All methods and animal studies were performed in accordance with the relevant guidelines and regulations of, and were approved by, the Institutional Animal Care and Use Committee of Sun Yat-sen (Zhongshan) University.

### Animals, cells and antisera preparation

Mature male and female Korean lamprey, *Eudontomyzon morii* were collected from the Yalu River of Dandong Valley in Liaoning province of northeast China. Adult lampreys (23–29 g, 23–28 cm) were respectively inoculated with 100 μg lipopolysaccharides (LPS, from *E. coli* 055:B5, Sigma) in 100 μl 0.9% NaCl or 100 μl 0.9% NaCl (control) via four intraperitoneal injections at 10 day intervals. Three to four days after the fourth immunization, blood was collected from tail-severed lampreys. The antisera were separated by centrifugation. The leucocytes were separated using medium of lymphocyte by centrifugation.

### Cloning the full-length cDNA of *NICIP* gene

All primer sequences are listed in SI Table 1. The total RNAs of the leucocytes were extracted with Trizol kit (Invitrogen), and converted to cDNA with a SMARTer™ RACE cDNA Amplification kit (Clontech Laboratories, Inc.). The 5′-Rapid amplification of cDNA ends (5′-RACE) and 3′-RACE were carried out by LA Taq DNA polymerase (Takara) and the gene specific primers. The amplified DNA fragments were cloned into a pGEM-T Easy Vector (Promega) for sequencing confirmation. The amino acid sequence of *NICIP* was analyzed using GeneDoc software, the MEGA 4 program, http://blast.ncbi.nlm.nih.gov/Blast.cgi and http://www.uniprot.org/uniprot. Conserved domains were analyzed at http://smart.embl-heidelberg.de/smart/set_mode.cgi and http://www.expasy.org/tools/scanprosite. The sequence of *NICIP* gene identified in lamprey had been submitted to GenBank sequence database. Protein three-dimensional structure prediction was submitted to http://www.sbg.bio.ic.ac.uk/phyre2/html/page.cgi?id=index.

### Construction of recombinant vectors

The extracellular segment containing V-set domain and full-length gene of NICIP were respectively cloned into pET-32a (+) and pEGFP-N1 vectors. The recombinant pET-32a-V-set-NICIP and pEGFP-N1-NICIP plasmids were identified by sequencing.

### Real-time PCR

The primer sequences of Real-time PCR are given in Table S1. Total RNA from various lamprey tissues was isolated using Trizol reagent (Invitrogen) and contaminating genomic DNA was removed by the treatment of RNase-free DNase (Promega). First-strand cDNA was synthesis with random primers (Takara) from 10 μg total RNA in 50 μl. RT-PCR was performed according to manufacturer’s protocol (Takara). Quantitative real-time PCR was performed using the Light Cycler 480 system (Roche). Each reaction had a total volume of 10 μl containing 1 μl cDNA, 5 μl SYBR^®^ Green Real-time PCR Master Mix (ToYoBo; QPK-201), 3.2 μl sterile purified water and 0.4 pmol of the appropriate forward and reverse primers. The following PCR program was used: 30 s at 95 °C, 35 cycles of 5 s at 95 °C, 10 s at 54 °C and 20 s at 72 °C. Subsequently, a melting curve analysis was performed, which consisted of 70 cycles of 10 s with a temperature increment of 0.5 °C per cycle starting at 60 °C. The cycle threshold (Ct) values were obtained. The relative expression of *NICIP* mRNA in tissues versus kidney was calculated using 2^−∆∆CT^. ∆Ct = Ct (*NICIP*) − Ct (*GAPDH*), ∆∆Ct = ∆Ct (Various tissue) − ∆Ct (Kidney). All tissues samples were performed in triplicates. The each kind of tissues was acquired from six lampreys, three lampreys were stimulated with LPS in 0.9% NaCl and three lampreys were simultaneously injected with 0.9% NaCl as control.

### Recombinant proteins expression and antibodies (Abs) production

To produce polyclonal Abs against pET-32a-V-set-NICIP, a recombinant plasmid of pET-32a-V-set-NICIP was transformed into *E. coli* BL21 and was selected using ampicillin. BL21 were grown overnight in Luria Betani broth at 37 °C until the optical density at 600 nm was between 0.4 to 0.6. Then, isopropyl-β-d-thiogalactopyranoside (1 mM) was added to the culture, which was then incubated at 37 °C for 3 h. The expressed recombinant proteins were affinity-purified by Ni^2+^ Sepharose(GE Healthcare) and analysed by sodium dodecyl sulphate-polyacrylamide gel electrophoresis (SDS-PAGE) (Fig. S1).

New Zealand white rabbits were immunized via multipoint intradermal injection for four times at 14 day intervals with V-set-NICIP recombinant proteins. For the first immunization, 500 μg of the recombinant protein antigen in 500 μl PBS (Phosphate Buffer Saline) was incorporated in an equal volume of Freund’s complete adjuvant (Sigma). For the three subsequent immunizations, 250 μg antigens in 500 μl PBS was emulsified with equal volume of Freund’s incomplete adjuvant (Sigma). On seven days after the fourth immunization, the sera of the immunized animals were collected and the polyclonal Abs were purified by recombinant proteins coupled CNBr-activated Sepharose (Fig. S2). The titer and specificity of the anti-NICIP Abs was confirmed by ELISA and western blotting (Fig. S3).

### Sorting of lamprey leucocytes and western blotting assays

Lamprey leucocytes were sorted into three discrete subpopulations (lymphocytes, monocytes, and granulocytes) based on the forward vs. 90u light-scattering profiles from flow cytometry. Cells were collected from each subpopulation using FACS Aria (BD Biosciences).

The sorted cells were digested with cell lysis buffer (20 mM Tris, pH 7.5, 150 mM NaCl, 1% Triton X-100, protease inhibitor Cocktail). Then the samples were analyzed by 10% denaturing SDS-PAGE and transferred to PVDF membrane (Millipore). The proteins were then subjected to western blotting analysis with rabbit anti-NICIP polyclonal Abs.

### Laser scanning confocal microscopy

The 293T cells were maintained in Dulbecco’s modiWed Eagle’s medium (Gibco) supplemented with 10% heat-inactivated fetal calf serum (Gibco).The cells cultured in 35 mm glass bottom culture dishes (MatTek Co.) were transfected with pEGFP and pEGFP-NICIP plasmids using a standard lipofectamine method (GIBCO/BRL). The DNA–lipid complex was removed after 8 h and the fresh medium was added. After 48 h, cell nuclei were stained with DAPI (Sigma). Fluorescent images were taken using A1 Nikon confocal laser microscope.

The lamprey leucocytes were separated using separation medium of mouse leucocyte (Haoyang Biotechnology, Tianjing, China). Separated leucocytes were washed three times using D-Hanks buffer (0.4 g/L KCl, 0.06 g/L KH_2_PO_4_, 8.0 g/L NaCl, 0.35 g/L NaHCO_3_, 0.132 g/L Na_2_HPO_4_·12H_2_O and 1.0 g/L D-glucose). Then the cells were stimulated with lamprey anti-LPS antisera and LPS antigen for 30 min at 4 °C. After washing three times, stimulated-leucocytes were incubated with rabbit anti-NICIP polyclonal Abs and mouse anti–VLRB monoclonal Abs (m-anti-VLRB mAbs, previously preparation in our laboratory). After washing three times, samples were then stained with got anti-rabbit IgG Alexa Fluor 488 (1:1000) and goat anti-mouse IgG Alexa Fluor 568 (1:1000). After washing three times, confocal images were obtained with an upright confocal microscope, Leica TCS-SP5.

### Flow cytometry analysis

The lamprey leucocytes were incubated with rabbit anti-NICIP polyclonal antibodies with 1:400 dilution, then the cells were stained with FITC-conjugated goat anti-rabbit IgG (Beijing Zhongshan Golden Bridge Biotechnology Co., LTD) and analyzed by flow cytometry (BD Biosciences).

### Co-immunoprecipitation and western blotting assays

The lamprey leucocytes were incubated with lamprey anti-LPS antisera with or without LPS antigen for 30 min at 4 °C. After washing three times, leucocytes were digested with cell lysis buffer (20 mM Tris. pH7.5, 150 mM NaCl, 1% Triton X-100, protease inhibitor Cocktail). The whole cell lysate were respectively incubated with 1 μg anti-VLRB mAb or anti-NICIP polyclonal antibodies and unrelated mouse or rabbit IgG antibodies for 2 hr at 4 °C. Protein G agarose was then added to each sample. The mixture was incubated at 4 °C for 4 hr, followed by centrifugation to collect the agarose with precipitated proteins. The samples were analyzed by 10% denaturing SDS–PAGE and transferred to PVDF membrane (Millipore). The proteins were then subjected to western blotting analysis with indicated antibodies.

The 293T cells were transfected with pEGFP-NICIP wt, pEGFP-NICIPY295F, pEGFP-NICIPY318F and pEGFP-NICIPY295F Y318F plasmids. After 48 h, the medium were replaced using lamprey anti-LPS antisera and LPS antigen, and transfected cells were stimulated for 30 min at 37 °C. Then, the transfected cells expressing NICIP–GFP fusion proteins were pretreated with sodium pervanadate, then digested with cell lysis buffer (20 mM Tris. pH7.5, 150 mM NaCl, 1% Triton X-100, sodium pyrophosphate, β-glycerophosphate, EDTA, Na_3_VO_4_, protease inhibitor Cocktail). The whole cell lysate were respectively incubated with 1 μg anti-GFP mouse mAb, anti-SHP-1 or anti-SHP-2 rabbit anti-human polyclonal antibodies (Abcam), PBS and control mouse or rabbit IgG antibodies for 2 hr at 4 °C^24^. Protein G agarose was then added to each sample. The mixture was incubated at 4 °C for 4 hr, followed by centrifugation to collect the agarose with precipitated proteins. The samples were analyzed by 10% SDS–PAGE and transferred to PVDF membrane (Millipore). The proteins were then subjected to western blotting analysis with indicated antibodies (anti-pTyr antibodies were purchased from Cell Signaling Technology).

### Statistical methods

SPSS software and Student’s two-sample t-test were used for statistical analysis.

## Additional Information

**How to cite this article**: Wu, F. *et al*. An inhibitory receptor of VLRB in the agnathan lamprey. *Sci. Rep.* **6**, 33760; doi: 10.1038/srep33760 (2016).

## Supplementary Material

Supplementary Information
