# Supplementary material for: An inhibitory receptor of VLRB in the agnathan lamprey
Source: Sci Rep. 2016 Oct 20;6:33760. doi: 10.1038/srep33760 (PMC5071834; doi:10.1038/srep33760)
Supplement: Supplementary Information [file srep33760-s1.doc]

Supplementary information

Title: An inhibitory receptor of VLRB in the agnathan lamprey

Authors: Fenfang Wu^1^,*, Liyong Chen^2^,*, Yong Ren^1^,*, Xiaojing Yang^1^, Tongzhou Yu^1^, Bo Feng^1^, Shangwu Chen^1^ and Anlong Xu^1, 3^

Table S1 Sequence of primers used for RACE and Real-time PCR

| Name | Sequence |
| --- | --- |
| 5’ RACE of *NICIP* |  |
| Sense | 5’-AAGCAGTGGTATCAACGCAGAGT-3’ |
| Anti-sense | 5’- CGTGGCAAGGTGGAGTG -3’ |
| 3’ RACE of *NICIP* |  |
| Sense | 5’- ACGCAACCAGTATGGGAAT -3’ |
| Anti-sense | 5’-CGCGGATCCTCCACTAGTGATTTCACTATAGG-3’ |

| Real Time PCR of *NICIP* |  |
| --- | --- |
| Sense | 5’-ACGCAACCAGTATGGGAAT-3’ |
| Anti-sense | 5’-CGTGGCAAGGTGGAGTG-3’ |
| *GAPDH* |  |
| Sense | 5’-GGGAGTAAACCACGAGAAGTA-3’ |
| Anti-sense | 5’-GGGTGTCGCCATTGAAGT-3’ |
| V-set domain cloning of *NICIP* |  |
| Sense | 5’- CGGGATCCTTGAAATCAATTGTTGGTG -3’ |
| Anti-sense | 5’- CCGCTCGAGTTATTCCACTGTAAGTCGCGTCCCT -3’ |
| Full-length genes cloning of *NICIP* |  |
| Sense | 5’- AGATCTCGAGACCATGGTCTCGCTGTTGTTT -3’ |
| Anti-sense | 5’- TCGAAGCTTGTTGTGCACGATGCTTGCAT -3’ |

Fig. S1

**a**

**b**


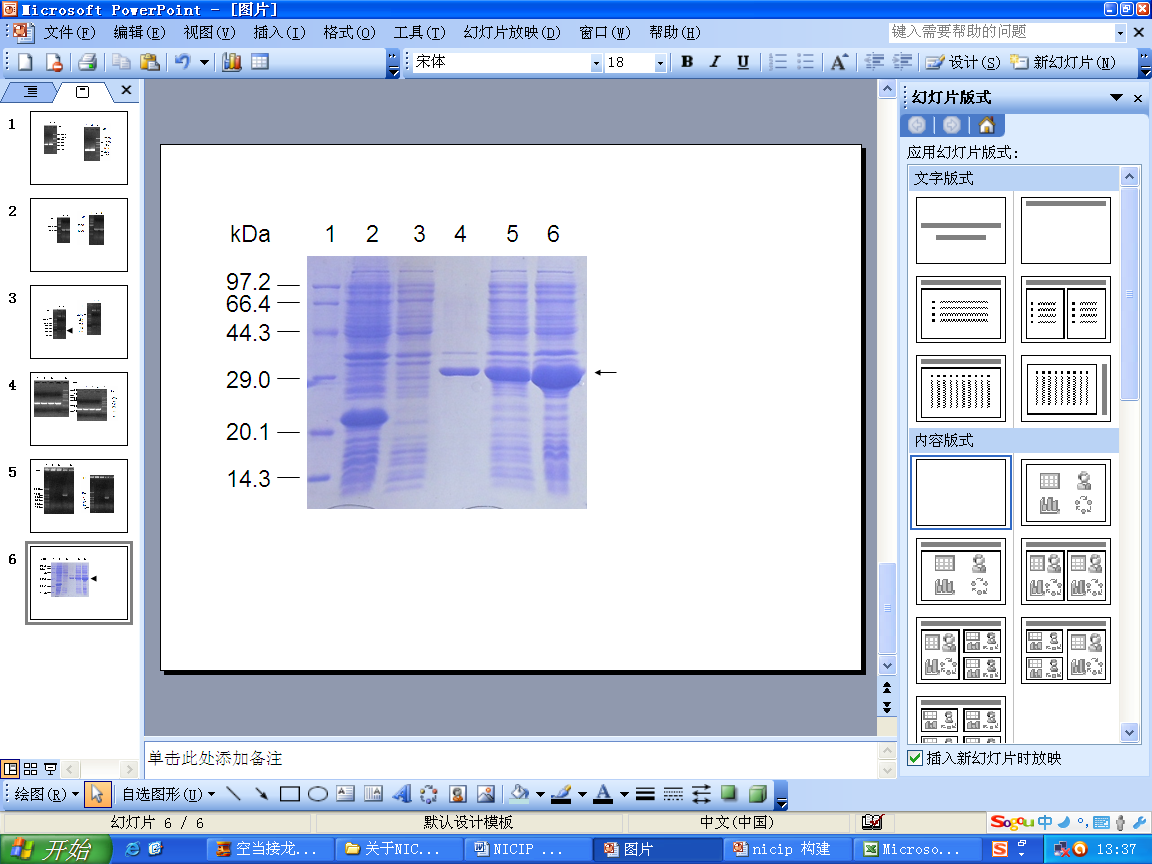

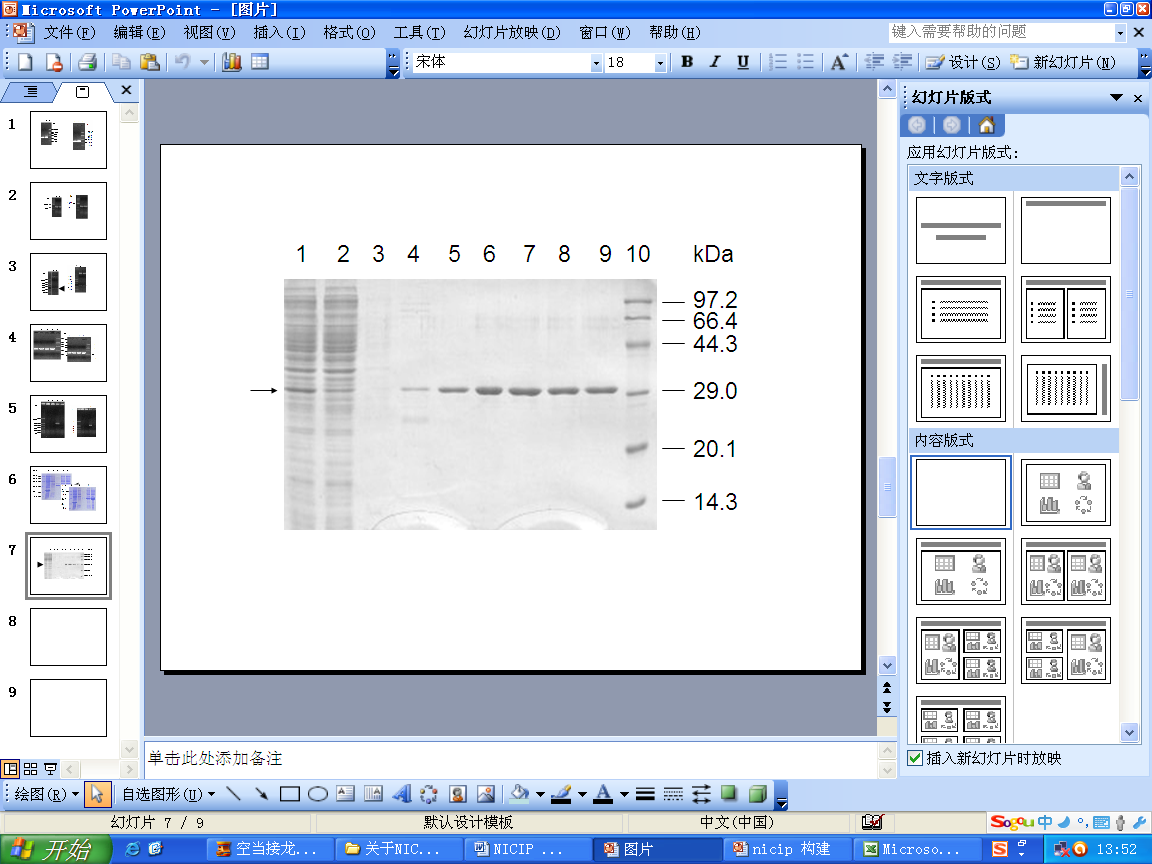


Figure S1. Expression and purification of recombinant protein. (a) Expression of recombinant protein in BL21 cells. Lane 1, protein marker; Lane 2, total protein of induced cells harbouring pET32a; Lane 3, total protein of un-induced cells harbouring pET32a-V-set-NICIP; Lane 4, precipitation from induced cells harbouring pET32a-V-set-NICIP after sonication；Lane 5, supernatant from induced cells harbouring pET32a-V-set-NICIP after sonication；Lane 6, total protein of induced cells harbouring pET32a-V-set-NICIP. (b) Purification of recombinant protein. Lane 1, supernatant from induced cells harbouring pET32a-V-set-NICIP after sonication；Lane 2, flowing through; Lane3~4: equilibrium; Lane5~9, elution by imidazole ,concentration gradient from 50mM, 100mM, 200mM, 300mM to 500mM; 10, protein marker.

Fig. S2


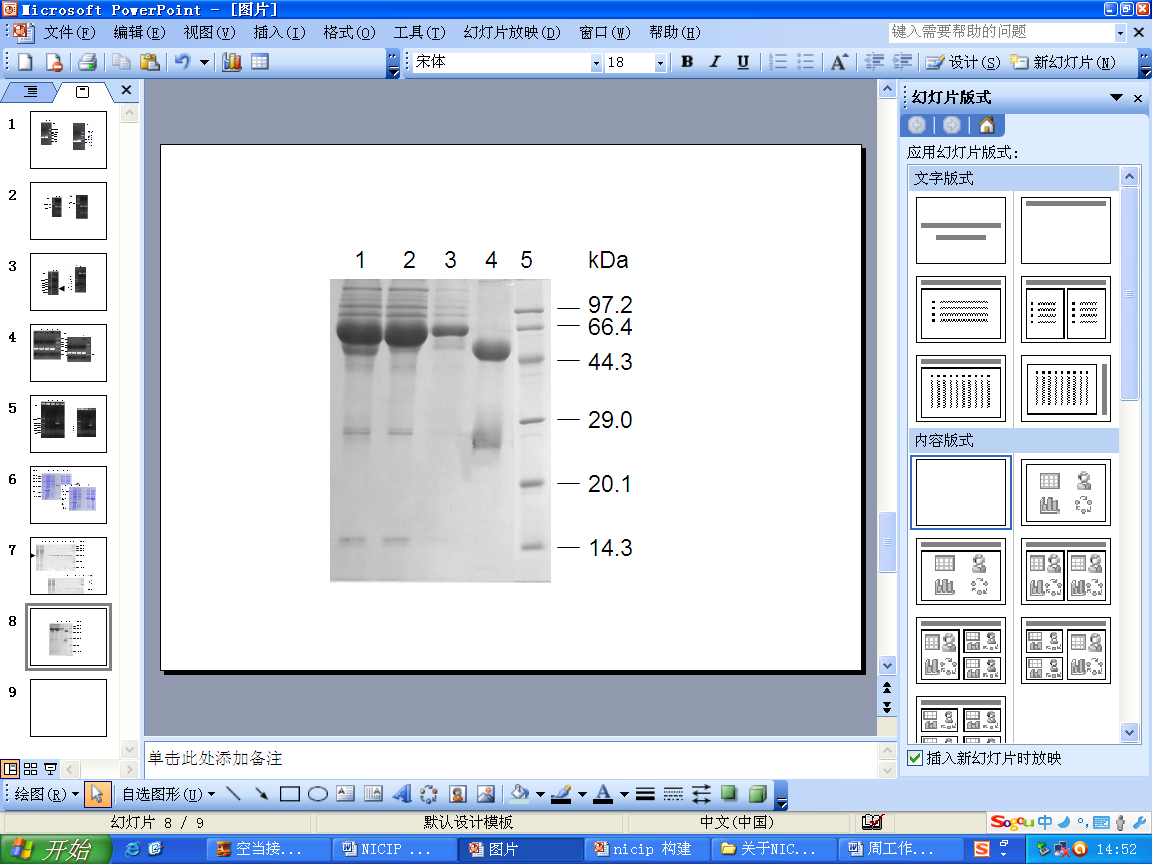


Figure S2. Purification of anti-V-set-NICIP polyclonal antibody

Lane 1~2, antiserum; Lane 3, equilibrium; Lane 4, elution; Lane 5, protein marker.

Fig. S3

**
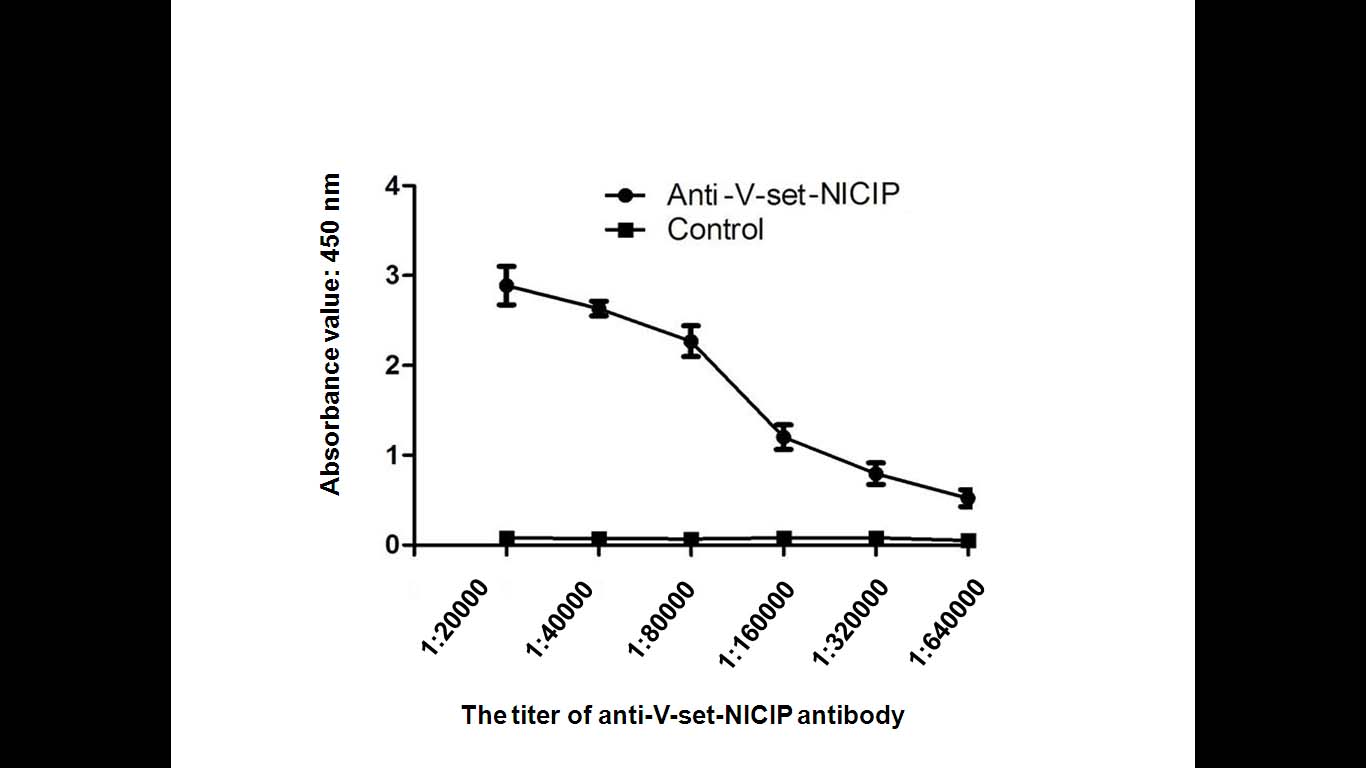
a** **b**

kDa

66.4

44.3


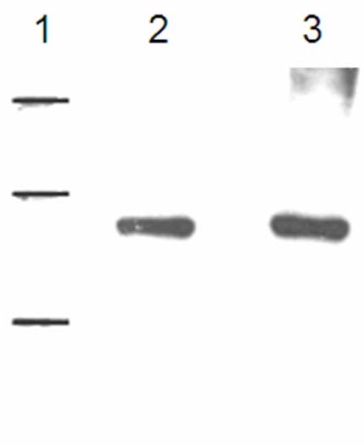


29.0

Figure S3. Identification of anti-V-set-NICIP polyclonal antibody

(a) The titer of anti-V-set-NICIP polyclonal antibody by ELISA assay. The control was no irrelevant rabbit Ig sample. (b) Specificity of anti-V-set-NICIP polyclonal antibody by western blotting assay. Loading sample, leukocyte lysate of lamprey. Lane 1, protein marker; Lane 2, the anti-V-set-NICIP polyclonal antibody was diluted to 1:4000; Lane 3, the anti-V-set-NICIP polyclonal antibody was diluted to 1:2000.
